# A Survey of Honey Bee Colony Losses in the U.S., Fall 2007 to Spring 2008

**DOI:** 10.1371/journal.pone.0004071

**Published:** 2008-12-30

**Authors:** Dennis vanEngelsdorp, Jerry Hayes, Robyn M. Underwood, Jeffery Pettis

**Affiliations:** 1 Pennsylvania Department of Agriculture, Bureau of Plant Industry–Apiculture, Harrisburg, Pennsylvania, United States of America; 2 Department of Entomology, The Pennsylvania State University, University Park, Pennsylvania, United States of America; 3 Florida Department of Agriculture, Bureau of Plant and Apiary Inspection, Apiary Inspection Section, Division of Plant Industry, Gainesville, Florida, United States of America; 4 United States Department of Agriculture (USDA)–ARS Bee Research Laboratory, Beltsville, Maryland, United States of America; University of Cambridge, United Kingdom

## Abstract

**Background:**

Honey bees are an essential component of modern agriculture. A recently recognized ailment, Colony Collapse Disorder (CCD), devastates colonies, leaving hives with a complete lack of bees, dead or alive. Up to now, estimates of honey bee population decline have not included losses occurring during the wintering period, thus underestimating actual colony mortality. Our survey quantifies the extent of colony losses in the United States over the winter of 2007–2008.

**Methodology/Principal Findings:**

Surveys were conducted to quantify and identify management factors (e.g. operation size, hive migration) that contribute to high colony losses in general and CCD symptoms in particular. Over 19% of the country's estimated 2.44 million colonies were surveyed. A total loss of 35.8% of colonies was recorded; an increase of 11.4% compared to last year. Operations that pollinated almonds lost, on average, the same number of colonies as those that did not. The 37.9% of operations that reported having at least some of their colonies die with a complete lack of bees had a total loss of 40.8% of colonies compared to the 17.1% loss reported by beekeepers without this symptom. Large operations were more likely to have this symptom suggesting that a contagious condition may be a causal factor. Sixty percent of all colonies that were reported dead in this survey died without dead bees, and thus possibly suffered from CCD. In PA, losses varied with region, indicating that ambient temperature over winter may be an important factor.

**Conclusions/Significance:**

Of utmost importance to understanding the recent losses and CCD is keeping track of losses over time and on a large geographic scale. Given that our surveys are representative of the losses across all beekeeping operations, between 0.75 and 1.00 million honey bee colonies are estimated to have died in the United States over the winter of 2007–2008. This article is an extensive survey of U.S. beekeepers across the continent, serving as a reference for comparison with future losses as well as providing guidance to future hypothesis-driven research on the causes of colony mortality.

## Introduction

Honey bees are an essential component to modern American agriculture. The value of honey bee pollination services to U.S. agriculture has been estimated to be greater than 14 million dollars [1] with their value topping $215 billion worldwide [2]. More than three-quarters of all flowering plants must be pollinated by an animal visitor; usually an insect [3]. In addition, it often takes several floral visits by pollinators to ensure maximum fruit set and quality [4]. Large acreages of pollinator-dependent crops, such as apples, almonds, blueberries, and cranberries, require managed pollinators to ensure production. The ability to easily move and manage honey bees, *Apis mellifera*, makes them ideal for this purpose. In all, it has been estimated that directly, and indirectly, one-third of the food we eat comes from honey bee pollination [5]. Honey bee health is challenged on many fronts [6]. Parasites, such as varroa mites (*Varroa destructor*), honey bee tracheal mites (*Acarapis woodi*) [5], fungal, bacterial and viral diseases, and kleptoparasites such as small hive beetles (*Aethina tumida*), many of which have been introduced over the last 20 years to the continental U.S., are all challenges faced by beekeepers. In 2006, a poorly understood phenomenon, Colony Collapse Disorder (CCD), resulted in widespread losses in the U.S. [7]. While viruses and fungal pathogens have been identified as good indicators of this condition, these pathogens, on their own, are not able to explain all losses, suggesting that honey bee colonies are suffering from compromised immune systems which pathogens are able to take advantage of. Pesticides, both those applied to field crops and to hives to control bee parasites, and beekeeping management have both been proposed as contributing to the honey bees' compromised immune systems [8].

Considering all the known and postential threats to honey bee colonies it is not surprising that honey bee populations have been declining over the last one half century. The National Agriculture Statistics Service (NASS) reported that there were 2.44 million honey-producing colonies in the United States as of February 2008 [9], down from 4.5 million in 1980, and 5.9 million in 1947 [4]. NASS numbers may underestimate the total number of managed colonies as they exclude colonies managed for pollination contracts only and do not include colonies that are managed by beekeepers owning fewer than 5 hives. This underrepresentation may be offset by the NASS practice of counting some colonies more than once; colonies that are moved to different states to produce honey are counted in each state's total and summed in total colony counts. Regardless of the exact accounting, the declining numbers of managed colonies is indisputable.

Unfortunately, NASS reports do not give an indication of annual winter losses. Commercial beekeepers have always relied on the ability to replace colonies lost in winter with new ones in the spring. Colonies that survive the winter quickly build their adult populations. Beekeepers can then “split” these colonies by removing half of the immature and adult bee population, introducing them into the equipment of a dead colony, and adding a new queen. This practice permits beekeepers to build their colony numbers back up by mid-summer even after suffering losses of 50% or more. Winter losses are, therefore, unrepresented in NASS figures unless beekeepers decide not to or are unable to replace winter losses. The survey described here is an attempt to quantify the extent of honey bee colony losses over the winter of 2007–2008.

High honey bee mortality appears to be widespread, as Canada [10], Germany, France, Great Britain, and the Netherlands have all reported elevated losses over the last several years [e.g. 11]. These losses are often poorly documented and epidemiological details, such as symptoms of death, extent of mortality, etc. are mostly non-existent. Such documentation, however, is critical for tracking trends and suggesting underlying causes of mortality. This study quantifies colony losses experienced in beekeeping operations in the United States between September 2007 and March 2008. It also attempts to identify factors, such as operation size, hive migration, etc., that may contribute to losses in general, and CCD in particular. We hope such findings will guide future hypothesis-driven research, which, in turn will help stem future colony losses.

## Methods

All members of the Apiary Inspectors of America (AIA) were asked to survey beekeepers in their states during the week of 23–30 March 2008. AIA members were asked to contact beekeepers by telephone that they felt were representative of their state's apiary industry, and to contact a minimum of 15 beekeepers: 5 part-time (1–50 colonies), 5 sideline (51–499 colonies), and 5 commercial (500+ colonies). They asked the following questions:

1. In what state and county do you keep your hives?
2. How many hives did you have alive in September 2007?
3. How many hives are alive now (March 2008)?
4. How many splits, increases, and/or colonies did you make/buy since September 2007?
5. Were your losses over this time period what you would consider to be normal?
6. What percentage of your hives that died had no dead bees in the hive or in the apiary?
7. To what do you attribute the cause of death for the hives that died?
8. What percentage of your hives did you send to CA for almond pollination?

Similarly, the United States Department of Agriculture (USDA)–Agricultural Research Service Beltsville Bee Research Lab conducted a survey of large commercial beekeepers, but their survey differed from the AIA survey in that it did not ask question #6. After the results were submitted, AIA and USDA surveyors were asked to report the number of beekeepers that refused to take the survey.

In addition, the survey questions were sent by e-mail to BEE_L, an internet mailing list, and to all Pennsylvania (PA) state local association presidents (n = 13) who were requested to send the questionnaire to all beekeepers on their e-mail distribution lists. The letter asked beekeepers to respond to a dedicated e-mail account. The results of three surveys, AIA, e-mail, and USDA, are reported here.

### Calculations

Total colony losses were calculated for each reporting operation, for the sum total of all respondents, and for various subgroup classifications. The mean of individual operation losses was calculated to determine the average loss among subgroups. Point estimates of the 95% Confidence Intervals (95% CI) were also calculated [12]. In cases where the total number of respondents for a reported group was less than 60, a normal distribution was not assumed and a t-distribution (based on n-1) was used to calculate the 95% CI [13].

Comparisons between total losses experienced by different groups of operations were conducted using the Chi Square test. Only significant results (P<0.05) are reported.

The total number of colonies lost with the symptom of no dead bees in the colony was calculated for individual operations by multiplying the number of colonies lost in an operation by the reported percentage lost without dead bees.

When calculating losses in individual states, colonies that were reported to be in more than one state during the period were counted multiple times; once in each listed state. This same practice is used by the National Agricultural Statistics Service when calculating the number of honey producing colonies in each state.

Response to the e-mail request was sparse for all states other than PA. The PA e-mail data were kept separate from the phone and USDA surveys, and grouped by county and then by climatic region as defined by the Pennsylvania State Climatologist (http://climate.met.psu.edu/data/state/pareg.php). Total losses in different regions were compared using the Chi Square test. Losses were also correlated with climate data downloaded from the same website.

## Results

### Total national losses

In all, 23 state apiarist offices assisted in conducting the phone survey in their respective states. States that did not participate in the survey lack inspection programs, lack the necessary resources for such endeavors, or felt that the timing was not appropriate for obtaining reliable information in their state. In total, the AIA surveyed 305 beekeeping operations, representing a total of 324,571 managed colonies in September 2007. This represents approximately 13.3% of the 2.44 million honey-producing colonies managed in the United States in 2007 [9]. The total loss reported over the surveyed period was 35.9% (95% CI: 30.5–41.3%) with an average loss of 31.0% (95% CI: 30.6–40.9%). In addition, the USDA surveyed 29 operations representing a total of 223,280 colonies in September of 2007. The total loss reported by the USDA survey was 36.8% (95% CI: 19.2–54.3%) with an average loss of 34.5% (95% CI: 16.4–52.6%). The two datasets were combined for the duration of the analyses after duplicate respondents were removed along with operations that did not provide essential information.

The combined dataset (AIA plus USDA) included 331 operations. The total number of colonies managed by these beekeepers in September 2007 was 474,336 representing 19.4% of the estimated 2.44 honey-producing colonies in the U.S. in the summer of 2007. The surveyed beekeepers reported having added a total of 81,501 new colonies to their operations between September 2007 and March 2008. In all, the total number of colonies living in March 2008 was 386,385. This represents a total loss of 35.8% (95% CI: 30.6–40.9%) and an average loss across all operations of 31.3% (95% CI: 7.4–54.1%). Should these surveys be representative of the losses across all operations, this suggests that between 0.75 and 1.00 million colonies died in the United States over the winter of 2007–2008.

Thirteen of the 22 surveyors responded to the request to report the number of beekeepers that refused to take the survey. Zero of 254 beekeepers refused, giving a 100% participation rate of individuals contacted directly by phone.

### Losses by operation classification (size, multi state, and CA almond pollinators)

When the respondents were classified by operation size there was no difference in either the total or average loss (Table 1). Operations that managed bees in more than one state did not have appreciably more losses than operations that managed bees in only one state (Table 2). In addition, operations that utilized some or all of their colonies for almond pollination in CA had average losses comparable to the average losses in operations that did not pollinate almonds. While the total losses reported by almond pollinators were elevated when compared to non-almond producers, the difference was not significant (Table 3).

**Table 1:** Average and total losses experienced by all responding beekeepers in the AIA and USDA surveys.

Operation Size	Number of Respondents	Colonies Managed in September 2007 Plus Increases	Average Loss % (95% CI)	Total Loss % (95% CI)
1 to 50	112	1,472	32.0 (23.3–40.6	33.2 (24.5–42.0)
51 to 500	94	17,211	29.1 (19.9–40.6)	31.2 (21.8–40.6)
500+	125	455,653	32.2 (24.4–40.4)	36.0 (27.6–44.4)

**Table 2:** Average and total losses experienced by all responding beekeepers in the AIA and USDA surveys who managed bees in one or more than one state.

More than One State	Number of Respondents	Colonies Managed in September 2007 Plus Increases	Average Loss % (95% CI)	Total Loss % (95% CI)
No	231	89,209	31.2 (25.2–37.2)	34.6 (28.7–40.6)
Yes	100	450,144	31.5 (22.3–40.6)	36.1 (25.7–46.4)

**Table 3:** Average and total losses experienced by all responding beekeepers in the AIA and USDA surveys who moved or did not move colonies into California almond groves for pollination.

Moved to CA	Number of Respondents	Colonies Managed in September 2007 Plus Increases	Average Loss % (95% CI)	Total Loss % (95% CI)
No	248	211,660	31.4 (25.6–37.2)	28.6 (22.9–34.2)
Yes	83	344,177	30.9 (20.9–40.8)	39.7 (29.2–50.3)

### Losses in operations reporting at least some CCD-like symptoms

One of the symptoms of CCD is the complete absence of bees in dead colonies or apiaries. This survey did not allow differentiation between true cases of CCD and colonies lost due to causes that share the “absence of dead bees” symptom. The 37.9% of operations (n = 102) that reported having at least some of their colonies die with this symptom and who reported the percentage of their losses with this symptom, had a significantly higher total loss of colonies (40.8%; 95% CI: 31.2–50.2%) than that experienced by operations that did not report this symptom (17.1%; 95% CI: 11.4–22.7%; χ^2^ = 3041, P<0.0001). At least 72.6% (n = 170) of all operations could not attribute any of their losses to CCD.

Large commercial operations were 4.5 and 1.8 times more likely to report having some of their colonies die without the presence of dead bees when compared to part-time and sideline beekeepers, respectively (Fisher's Exact test, P<0.0001; Table 4). In all, 60.0% of colonies that died had no dead bees in the colony, with the percentage of colonies lost with this symptom higher among larger operations (Table 4).

**Table 4:** CCD-like symptom reported in the AIA and USDA surveys.

Operation Size	Number of Respondents	% of Respondents with Some Incidence of No Dead Bees	Number of Colonies Lost	% of Colonies Lost Without Dead Bees
1 to 50	95	14.7	490	13.5
51 to 500	84	34.5	5,135	32.5
500+	93	63.4	111,499	61.5
**Total**	**272**	**37.5**	**117,124**	**60.0**

### Normal losses

Three hundred and eighteen respondents answered the question as to whether they felt their losses were “normal” or not. Several respondents (n = 17) answered, “no–losses were less than normal”. These respondents had their answers changed to “yes” as the intent of the question was to identify beekeeper perception of acceptable and non-acceptable losses. Thirty-eight percent of all respondents felt their losses were not normal, having an average loss of 47.8% (95% CI: 29.0–56.7%) as compared to those experiencing what they felt were normal losses that had an average loss of 21.7% (95% CI: 15.9–27.5%).

### Perceived cause of losses

Respondents were asked to identify why they thought their colonies died. Of the 229 persons responding to this question, 201 listed only one factor as responsible for their losses. Those reporting more than one reason were counted multiple times. The top five reasons given to explain death were poor quality queens, starvation, mites, CCD, and weather (Table 5). The total loss (48.2%) experienced by the group claiming CCD was the cause of mortality was higher than any other group (Table 5). Other factors that were mentioned, but were reported by fewer than 8% of respondents were management (7.8%), weak colonies in the fall (7.4%), Nosema/dysentery (4.4%), nutrition (3.5%), stress (3.1%), viruses (3.1%), and pesticides (2.6%). All other factors, including small hive beetle, American foul brood, bears, and transportation were reported by less than 1% of respondents.

**Table 5:** The five most commonly mentioned suspected causes of colony losses (total n = 229 operations) in the AIA and USDA surveys.

Cause	Rank	% of Operations Reporting	Number of Colonies Managed	Total Loss % (95% CI)
**Poor Queen**	1	31	145,655	18.5 (13.5–23.6)
**Starvation**	2	28	34,145	19.8 (19.6–25.0)
**Mites**	3	24	143,463	31.7 (25.6–37.2)
**CCD**	4	9	150,870	48.2 (41.7–54.8)
**Weather**	5	9	25,180	24.4 (18.8–30.0)

### Losses by state

**Figure 1:**
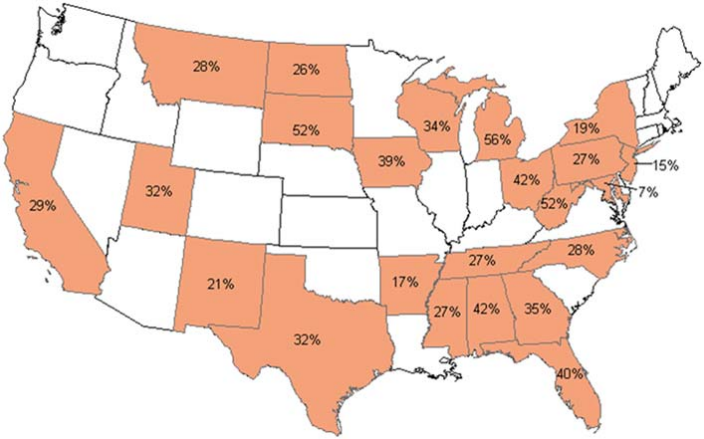
Total colony loss (%) by state.

Considerable variability in total and average losses was reported from the various states (Table 6; Figure 1). Only those states that had more than 6 respondents are included (disqualifying LA and SC from this portion of the analysis). In cases where a beekeeper kept bees in more than one state, the total losses are included in all states in which bees were kept. The number of beekeepers that were counted in more than one state, and the total percentage of hives they managed in the respective states are presented (Table 6).

**Table 6:** State by state losses reported in the AIA and USDA surveys of states having 6 or more respondents.

State	All Respondents			Operations Reported in Multiple States	
	Oper. (N)	Number colonies (September 2007+Increases)	Total Loss (CI 95%)	n	% total col
AL	15	5,329	42.0 (14.8–69.1)	-	-
AR	20	17,456	17.4 (0–35.2)	-	-
CA	36	200,704	29.3 (13.9–44.6)	31	90
FL	23	75,297	40.0 (18.9–61.2)	8	65
GA	15	53,956	34.5 (8.1–60.8)	-	-
IA	10	685	39.4 (4.4–74.3)	-	-
MD	13	4,080	7.3 (0–22.2)	-	-
MI	15	7,302	56.2 (28.7–82.7	1	8
MS	14	9,145	26.7 (3.9–49.9)	-	-
MT	13	62,865	27.7 (0.6–52.3	9	99
NC	16	7,866	27.5 (3.5–51.4	-	-
ND	18	113,842	25.6 (3.5–51.4)	15	99
NJ	15	23,532	15.1 (0–34.8)	3	88
NM	7	5,610	20.5 (0–57.8)	3	21
NY	9	29,035	18.8 (0–49.6)	3	62
OH	9	1,565	42.4 (34.0–81.3)	-	-
PA	32	16,141	27.0 (11.0–43.0)	3	56
SD	18	119,404	52.0 (27.0–76.9)	3	20
TN	8	516	26.5 (0–62.6)	-	-
TX	9	57,275	32.3 (0–69.2)	9	100
UT	25	17,104	32.3 (8.1–69.2)	-	-
WI	15	8,022	33.8 (7.6–60.0)	1	52
WV	17	3,786	51. 6(25.0–78.2)	1	38

### Losses by climatic region in Pennsylvania

**Figure 2:**
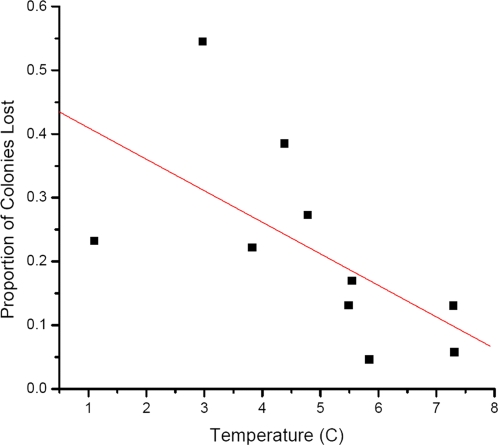
Correlation of colony losses reported in the e-mail survey in PA with average ambient temperature between September 2007 and March 2008.

A total of 174 respondents answered the e-mail survey, with a total of 160 coming from PA. As there were insufficient responses from other states to make meaningful comparisons, only PA data were included in this comparison. Operations were divided into categories by climatic region as mentioned previously. Data on monthly temperature and precipitation for each of the 10 regions were obtained. Average precipitation did not affect the proportion of colonies lost in a region (R = 0.07739, P = 0.8317). However, there was a weak correlation with average temperature (R = −0.61758, P = 0.0571). Regions with relatively lower average temperatures had higher colony losses (Figure 2).

## Discussion

Honey bee losses continue to increase in the U.S. as demonstrated by the losses reported here and in the AIA survey from 2007 [14]. It is difficult to partition colony losses into discrete loss categories with the exception of starvation. Part of the difficulty arises from the fact that most colonies suffer from multiple pests and diseases and the role of each is not easily defined or quantified without detailed longitudinal epidemiological studies. Further, different threats to honey bee health may act synergistically. For instance the presence of varroa mite does not necessarily act alone to impact bee health [15]. Prior to the introduction of varroa mites into the U.S., beekeepers reported 5–10% winter losses. These losses rose to 15–25% with the introduction of varroa and tracheal mites in the mid-1980s [4]. Here we report a second year of greater than 30% colony loss. The total loss of colonies increased from 31.8% during the winter of 2006–2007 to 35.9% over the winter 2007–2008 even as average operational losses decreased (37.6% and 31.0%, respectively) [14]. This apparent discrepancy is likely due to relatively higher total loss by larger operations (Table 1).

Commercial beekeepers are in tune with honey flows, the health status of their colonies and experience informs them what colony losses to expect in their specific region. Those beekeepers with multigenerational knowledge and experience can comment with authority on what “normal” losses are. Respondents to this survey were less likely to think their losses were “not normal” when compared to last year's survey [14]. During this survey, respondents reporting normal losses lost an average of 21.7% as compared to an average “normal” loss of 15.9% as reported previously. This suggests that beekeepers are beginning to expect more losses and accept higher mortality than they have in the past.

Stress associated with the movement of bees has been suggested as an underlying cause for increased mortality [8]. However, while total losses tended to be higher among California almond pollinators, the average operational losses in this group (30.9%) was no different than non-almond pollinating (31.4%) operations. This discrepancy can be explained by the exceptional high losses suffered by some of the larger operations surveyed.

The main symptom of CCD, a lack of dead bees present in hives from dead colonies, was more likely reported by beekeepers with large operations (Table 4). This suggests that a highly contagious condition may be responsible for this symptom, a problem that would be compounded by the crowded conditions that often exist in commercial beekeeping operations as they move colonies to and from pollination or honey-producing sites. One must keep in mind, as well, that large operations are generally found in agricultural settings where they are exposed to agricultural chemicals in all their forms and their diet may be incomplete from the monocrop situation they are often forced to feed upon. These factors may also contribute to the problem of CCD.

Of the perceived causes of losses starvation and poor queens were the most commonly identified. This is surprising, as both are manageable threats, suggesting a misdiagnosis of problems, a need to change management practices, and/or improved extension delivery methods.

A common practice in epidemiology is to look for spatial patterns to the occurrence of a disease or syndrome. With honey bee colonies making multiple moves around the country it is difficult to assign a colony loss to one region of the country. Losses were assigned to a specific state by the beekeeper and total losses varied by state (Figure 1; Table 6) with no discernable pattern. Within PA, however, losses did vary by region. In particular, regions with lower average temperatures experienced higher losses (Figure 2). This may be due to the direct effects of ambient temperature, or to the fact that more feed is necessary for nest homeostasis when it is colder, leading to starvation if feed becomes scarce.

This report documents colony losses in the U.S. While it remains difficult to accurately partition colony losses into discrete causes, our survey does indicate that one CCD symptom, the lack of dead bees in dead hives, is more common in larger operations, and operations reporting this condition had significantly higher losses than those that did not. This suggests that a contagious condition may be responsible for CCD. Continued surveys coupled with sample analysis from dead and dying colonies should provide clues as to the underlying cause increased rates of hive mortality in the U.S.
